# Comparison of EBT and EBT3 RadioChromic Film Usage in Parotid Cancer Radiotherapy

**Published:** 2016-03-01

**Authors:** M.T. Bahreyni Toossi, F. Khorshidi, M. Ghorbani, N. Mohamadian, D. Davenport

**Affiliations:** 1 Medical Physics Research Center, Mashhad University of Medical Sciences, Mashhad, Iran; 2 Comprehensive Cancer Centers of Nevada, Las Vegas, Nevada, USA

**Keywords:** RadioChromic film, EBT, EBT3, Radiotherapy, Parotid cancer

## Abstract

**Background:**

EBT and EBT3 radioChromic films have been used in radiotherapy dosimetry for years.

**Objective:**

The aim of the current study is to compare EBT and EBT3 radioChromic films in dosimetry of radiotherapy fields for treatment of parotid cancer.

**Methods:**

Calibrations of EBT and EBT3 films were performed with identical setups using a 6 MV photon beam of a Siemens Primus linac. Skin dose was measured at different points in the right anterior oblique (RAO) and right posterior oblique (RPO) fields by EBT and EBT3 films on a RANDO phantom.

**Results:**

While dosimetry was performed with the same conditions for the two film types for calibration and in phantom in parotid cancer radiotherapy, the measured net optical density (NOD) in EBT film was found to be higher than that from EBT3 film. The minimum difference between these two films under calibration conditions was about 2.9% (for 0.2 Gy) with a maximum difference of 35.5% (for 0.5 Gy). In the therapeutic fields of parotid cancer radiotherapy at different points, the measured dose from EBT film was higher than the EBT3 film. In these fields the minimum and maximum measured dose differences were 16.0% and 25.5%, respectively.

**Conclusion:**

EBT film demonstrates higher NOD than EBT3 film. This effect may be related to the higher sensitivity of EBT film over EBT3 film. However, the obtained dose differences between these two films in low dose range can be due to the differences in fitting functions applied following the calibration process.

## Introduction

Cancer is a general health problem in the United States of America and the world. One fourth of the death rate in the USA is due to cancer. The American Cancer Society reports new cases of cancer and the death rate due to those cases of cancers every year. It is anticipated that in the year 2014 there were be 1,665,540 new cases of cancer and 585,720 deaths as a result of them in the USA[1]. Since X-ray was discovered in 1895, ionizing rays have played an important role in medicine. Nowadays a wide range of different particles from photons, electrons, protons, and carbon ions, with an energy range of 10 keV to 100 MeV, are used in the field of imaging and therapy. Detection of these particles is necessary, not only to have successful diagnostic imaging, but also for ensuring that all units demonstrate reproducibility and the safety of the staff[2]. Radiotherapy is a form of cancer treatment method which can be effective to prevent the recurrence of many types of cancer. Since the radiation doses are not only delivered to the tumors, there are many types of toxicity for the adjacent normal structures[3]. The purpose of new therapy methods is to increase the effectiveness of treatment and to reduce toxicity.

The experimental results obtained from various dosimetric methods in different therapy departments reveal that frequently the delivered dose to the target is less than the prescribed dose. The reason for errors in dosimetry is partially related to the method of dosimetry[4]. Ionization chambers and semiconductor materials do not provide an appropriate spatial resolution for many measurements required for treatment planning in radiation therapy. Thermoluminescent dosimetry, even with small dimensions, presents unique dosimetric difficulties and is time consuming.

An alternative dosimetric technique which can be used in radiation dosimetry is silver halide radiographic films. Measurement of ionization radiation with use of silver halide radiographic films is difficult due to the existence of silver in these films. They show large energy dependency for photon energies in the range of 10 to 200 keV. The energy absorption characteristics of radiographic films are not directly equivalent to soft tissues. Other deficiencies of radiographic film are sensitivity to ambient light and the need for chemical processing is cost and space prohibitive. Such points inspire a search for a radiation dosimeter with higher spatial resolution with less difficulties and acceptable accuracy in absorbed dose measurement. RadioChromic films demonstrate some of these characteristics[5]. The introduction of radioChromic films based on polydiacetylene has solved some of the stated problems related to conventional two dimensional radiation detectors. Exhibiting characteristics such as high spatial resolution, low energy dependency for a wide range of energies used in radiation therapy, and near tissue equivalence makes such film suitable for measurement of dose in radiation fields with high dose gradients. These films are almost insensitive to visible light which makes them uniquely easy to use for processing, storage, and measurements in a room with normal light. RadioChromic films experience color change directly and do not need chemical processing. At their advent, Gafchromic films were used for industrial purposes. The sensitive layer of these films has a thickness of 6 micrometers. These semi-sensitive films are suitable for measurement of most high doses in the range of 50 Gy to 2500 Gy. They have been used for years for clinical dosimetry investigations under the name HD-810.

The HD-810 model was originally developed for use in radiation dosimetry. In the following years, the MD-55 model was produced with higher sensitivity. Development and progress of radioChromic films led to the Gafchromic EBT (External Beam Therapy) film model which was designed as a substitute for silver halide radiography films in quality assurance systems of intensity modulated radiation therapy[6]. In 2011, International Specialty Products (ISP) produced a new generation of films called Gafchromic EBT3 film. The active layer of EBT3 film is similar to EBT2 but its structure is symmetrical which prevents probable errors in measurement of optical density and also prohibits the fringe artifact formation[7].

Bilge et al[8] compared the surface dose from 6 MV and 18 MV photons with EBT film and compared the results with those from a parallel plate ionization chamber. The agreement between these two dosimeters for 6 MV photons was ±5% and for 18 MV photons was ±3%. Reinhardt et al[9] evaluated EBT3 films’ characteristics such as sensitivity, read out orientation, and post-exposure darkening. EBT3 film’s net optical density (NOD) response to photon and proton beams was investigated and compared with EBT2 film. Quenching effects in the proton’s Bragg peak area were also investigated for both films. Dosimetric performance of EBT2 and EBT3 were shown to be the same, thus EBT3 can be used for dose confirmation in IMRT similar to EBT2. However, for evaluation and confirmation of dose in proton therapy, the Bragg peak region must also be considered. Research was performed by Moylan et al[7] to compare EBT2 and EBT3 films for the film size, scanning condition, film model, and region of interest (ROI) size points of view. Another study was also performed by Carrasco et al[10] to compare EBT and EBT2 films for sensitivity to light, scanning direction, scanning dependency on both sides of the films, if scan results are repeatable, dependency on the location of the film in the scanner, time intervals between exposure and scanning, and if the films are homogenous. In another study, Brown et al[11] compared the dose response curve to synchrotron monoenergetic X ray of EBT, EBT2, and EBT3 films. However to the best of our knowledge, dosimetry characteristics in real clinical conditions were not compared for EBT and EBT3 films. The aim of this study is to compare dosimetry results of Gafchromic EBT and EBT3 films in skin dose measurement on a RANDO phantom in parotid cancer radiotherapy.

## Material and Methods

### Structure of EBT and EBT3 Films

**Figure 1:**
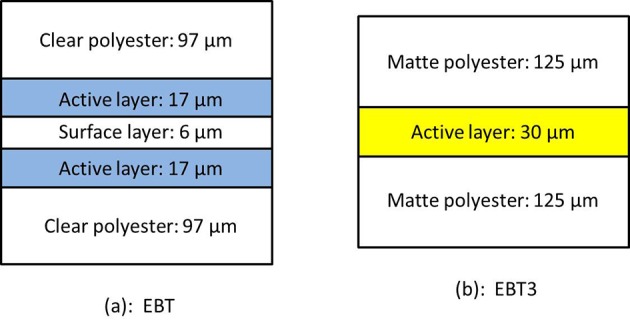
Schematic structure of EBT and EBT3 RadioChromic films

In this study EBT and EBT3 films have been used. These films are produced by ISP company which introduced EBT film in 2004 and EBT3 in 2011. EBT film was developed as a more sensitive film than the previous ones and its structure is more complicated than other previous Gafchromic films. Figure 1 (part a) illustrates that EBT film is made up of two active layers which are 17 micrometers thick. These two layers are separated by a 6 micrometer superficial layer. The whole structure is sandwiched between two polyester sheets which are 97 micrometers in thickness[12]. As shown in Figure 1 (part b) Gafchromic EBT3 film is made of a 30 micrometer active layer in the center and two 125 micrometer polyester layers on the sides of the active layer. Due to the symmetry of this film there is no front or back side for the film and both sides have identical scanning light conditions[13].

### Calibration of EBT and EBT3 radioChromic films

In order to calibrate Gafchromic EBT film, twenty pieces measuring 2 × 3 cm^2^ were cut from a single film sheet (lot number of 34351-05). In order to avoid any mistake in setting up the orientation of the films in the scanning process, the cutting was performed in a way that the length of these small pieces (3 cm) were along the length of the film sheet. These pieces were divided in 10 groups of two pieces with each piece allocated an identification code. In order to measure the background optical density (pixel value before irradiation), these films were scanned by a 1000XL Pro Microtek scanner before irradiation. Half an hour before reading each film, the scanner was turned on to warm up and stabilize. The films were scanned in transmission mode with a resolution of 100 dots per inch (dpi) and 48-bit red green blue (RGB) color mode. No color correction was applied and the scanning was performed in portrait direction and at the bottom of the scanner’s bed. Images obtained from scanning were saved non-zipped with tagged image file format (TIFF). In order to reduce the noise effects of the scanner, each piece of film was scanned three times. This scanner uses Microtek ScanWizard Pro software (version V7.041) for scanning. After scanning, each group was placed at 10 cm depth in the center of 10×10 cm^2^ radiation field in a PTW Solid Water phantom with dimensions of 30 × 30 × 20 cm^3^. The films were irradiated by a Siemens Primus linear accelerator in Reza Radiotherapy Oncology Center (in Mashhad) by using a 6 MV photon beam with 0.2, 0.5, 0.75, 1, 1.25, 1.5, 1.75, 2.5, 3.5, and 5 Gy doses. Irradiation of these films was performed at source to surface distance (SSD) of 100 cm. 36 hours after irradiation all film pieces were scanned by the Microtek 1000XL scanner similar to the procedure for scanning of background. NOD was calculated according to the formula:

NOD=OD_Cal_-OD_Back_=-(log_10_(P_Cal_)-log_10_(P_Back_))   (1)

OD_Cal_ is the calibration optical density, OD_Back_ is background optical density, P_Cal _is calibration pixel value and P_Back_ is background pixel value. The average amount of pixel value related to each scan in the red channel was calculated by using MATLAB (version 7.11.0.584, The Math Works Inc., Natwick, MA) software. Pixels located near the edges were excluded and the central part of the film with approximate size of 1 × 1 cm^2^ was considered in calculation of the average pixel value. The average pixel value was calculated from 3 separate scans for each piece of film. Dose (Gy) versus NOD was plotted and MATLAB software was used to fit power and exponential functions for EBT and EBT3 films.

Calibration condition of EBT3 film (with lot number of A04011301) was performed with the same procedure but the fitting curve was an exponential function for the EBT3 film.

### Measurement of dose in a Solid Water phantom 

An experiment was designed and performed in order to evaluate the radioChromic film for dose measurement in the build-up region. The results were compared with those from dosimetry measured with a Semiflex ionization chamber (Siemens, Germany). The method was to cut an EBT film sheet from the same film pack used for calibration to 2×3 cm^2^ pieces. The film pieces were scanned by the 1000XL Pro Microtek scanner for calculation of background optical density before irradiation. These pieces were located in the depths of 0, 0.1, 1.5, 2, 4, 10 cm of a PTW Solid Water phantom (RW3, Siemens, Germany)  with dimensions of 30 × 30 cm^2^ in a 10 × 10 cm^2 ^radiation field at SSD=100 cm. The films were irradiated in Reza Radiotherapy Oncology Center with prescription of 100 cGy to depth of 10 cm (equal to 100 MU) by 6 MV photons of a Siemens Primus linear accelerator. Reading stages of these pieces were performed in a similar method to calibration condition. Depth dose results from Gafchromic EBT film were compared to those results from Semiflex ionization chamber (Nominal sensitive volume 0.125 mm^3^, Germany). This test was repeated for EBT3 film under the same conditions.

### Treatment planning for parotid cancer on RANDO phantom

The following procedure outlines the creation of the treatment plan used in this study for determination of skin dose measurement. CT images were taken from a male RANDO Alderson phantom (The Phantom Laboratory, NY, USA) [14]. The phantom has 175 cm height and 73.5 kg weight. The CT imaging was performed using a Siemens SOMATOM Emotion Due CT scanner available at Reza Radiotherapy Oncology Center (Mashhad, Iran) with a slice thickness 0.5 cm. Treatment planning of parotid cancer was performed using Prowess Panther treatment planning system (Siemens, Germany). The plan was designed by an oncologist and included two oblique wedged fields: a right anterior oblique (RAO) and a right posterior oblique (RPO). The characteristics of this treatment planning are depicted in Table 1.

**Table 1:** Detailed information on RAO and RPO fields in treatment of parotid cancer.

	**RAO field**	**RPO field**
**SSD (cm)**	98.0	98.0
** Field size (cm^2^) **	6.0×8.4	6.0×8.4
**Collimator angle (º)**	10.0	350.0
**Gantry angle (º)**	325	235.0
**Wedge name**	3RW30	4RW30
**Wedge orientation**	X2	X1
**Wedge factor**	0.516	0.516

### Skin dose measurement in parotid cancer treatment

**Figure 2:**
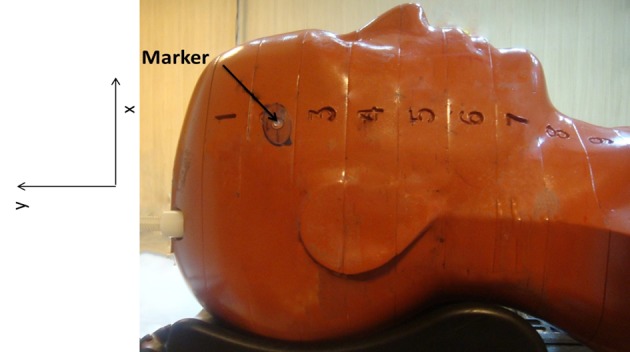
RANDO phantom and the position of the marker used in skin dosimetry of parotid cancer

**Figure 3:**
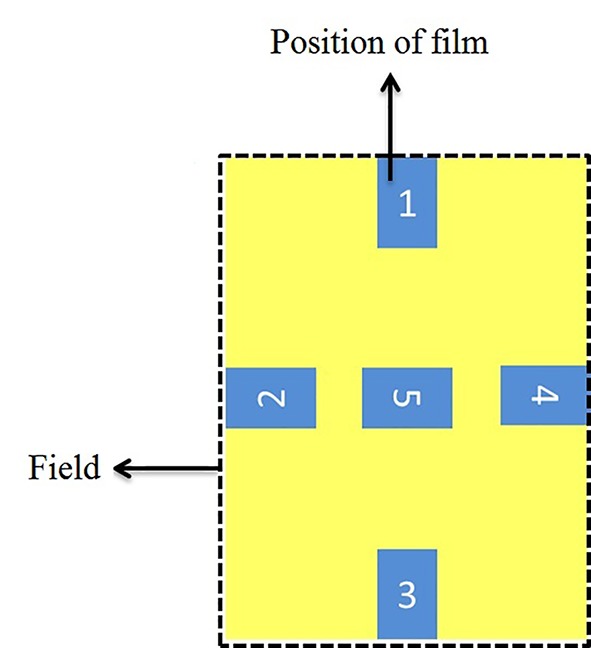
Schematic positions of the film pieces in the RAO and RPO fields

The previously described treatment plan was utilized for comparing the dosimetry results from EBT and EBT3 films.  The radiation marker and the head of the RANDO phantom are shown in Figure 2. Additionally, the positions of the film pieces in the RAO and RPO fields are schematically shown in Figure 3. Five points per field were determined for skin dose measurement. One point was considered on the center of the field and the rest of the points were considered in almost symmetrical positions on the sides. The coordinates of the measurement points in the RAO field relative to the radiation marker in centimeters are as follows: position 1: (*X*=-2.8, *Y*=-1.6); position 2: (*X*=-6.5, *Y*=-5.5); position 3: (*X*=-2.8, *Y*=-9.8); position 4: (*X*=1.7, *Y*=-5.5) and position 5: (*X*=-3, *Y*=-5.5).

The coordinates (in terms of cm) of the measurement points relative to the radiation marker in the RPO field are as follows:

Position 1: (*X*=-7, *Y*=-5.6); position 2: (*X*=-6.5, *Y*=-5.5); position 3: (*X*=-2.7, *Y*=-10); position 4: (*X*=1.5, *Y*=-5.4) and position 5: (*X*= -2.7, *Y*= -5.5).

One EBT film sheet was taken from the same pack used in calibration step with lot number of 34351-05. This film sheet was cut into 2 × 3 cm^2^ pieces. The pieces were divided in five groups, each containing three pieces. The pieces were scanned in order to calculate the background optical density before irradiation under calibration conditions. Each film piece in each group was stuck to the certain point on the field (positions from 1 to 5). The measurement described above was repeated three times for each group using the three films in that group. The RAO field was irradiated to a dmax dose of 1.014 Gy and RPO field to 1.037 Gy by a Seimens Primus linear accelerator. The film pieces were scanned 36 hours after the irradiations according to calibration conditions. Using MATLAB software under the calibration conditions, the NOD related to each measurement point was calculated and then by using the calibration function for each film, the related dose for each measurement point was calculated. Dose measurement conditions for EBT3 film was the same as the EBT and in all the cases the percentage difference between the value (NOD or dose) obtained from Gafchromic EBT and EBT3 films was calculated according to the following:

Percent Difference(%)=((value from EBT-value from EBT3)/(value from EBT)×100)    (2) 

## Results

### Calibration of EBT and EBT3 radioChromic films

**Figure 4:**
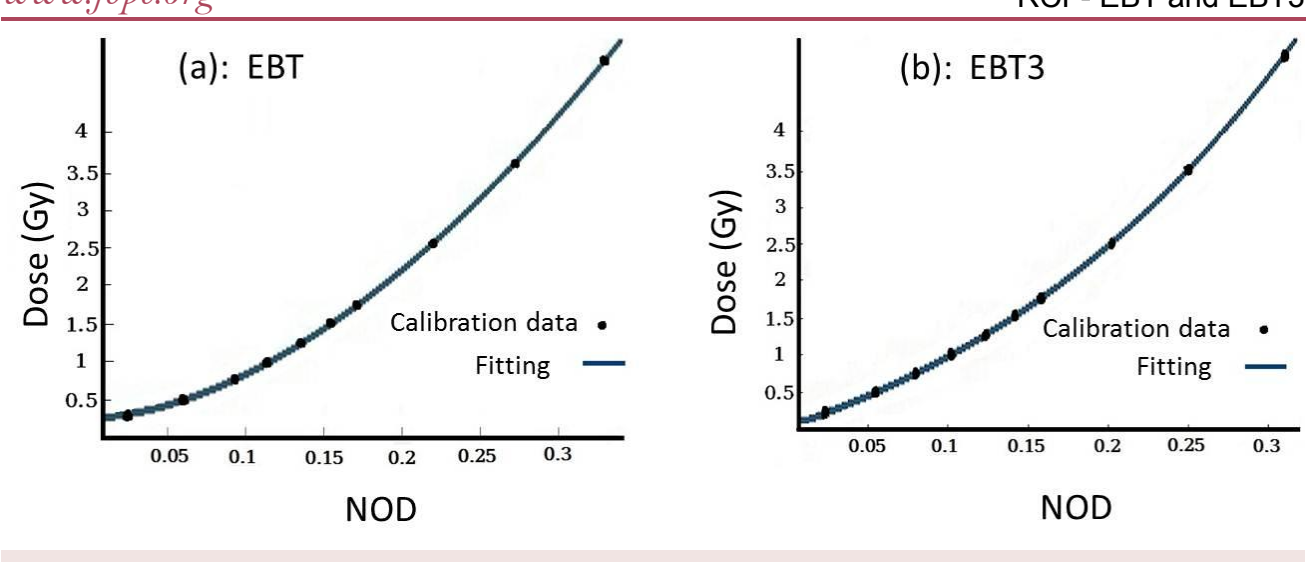
Calibration curve for EBT and EBT3 films

In Table 2, the NOD from the calibration process of EBT and EBT3 and the percentage differences between NOD from both films are listed. Figure 4 (part a) depicts the EBT film calibration curve. The fitting formula for dose in Gy through NOD for EBT film is according to equation:

*D*=33.45NOD^1.765^+0.2389         R^2^=0.9975        (3)

**Table 2:** NOD obtained from calibration process for EBT and EBT3 radioChromic films and the percentage dose differences (%).

**Delivered dose (Gy)**	**NOD**		**Diff. (%)**
	**EBT**	**EBT3**	
0.2	0.0241	0.0233	2.92
0.5	0.0592	0.0382	35.47
0.75	0.0930	0.0798	14.19
1.0	0.1127	0.1019	9.58
1.25	0.1355	0.1232	9.08
1.5	0.1540	0.1418	7.92
1.75	0.1702	0.1582	7.05
2.5	0.2204	0.2024	8.17
3.5	0.2743	0.2492	9.15
5.0	0.3275	0.3097	5.44

Figure 4 (part b) shows the calibration curve related to Gafchromic EBT3 film. The measured dose in terms of Gy can be calculated through measured NOD by formula 4:

*D*=75.57e^2.609NOD^-75.54e^2.513NOD^       R^2^=0.9999    (4)

### Dose measurement in Solid Water Phantom

NOD was calculated from radioChromic EBT and EBT3 films for measurements at different depths in a Solid Water Phantom and the percentage differences (%) of these values are listed in Table 3. Dose values (Gy) obtained from radioChromic EBT and EBT3 films at different depths in a Solid Water Phantom are expressed in Table 4. These values are compared with dosimetry results from the Semiflex ionization chamber and the percentage difference (%) between dose results from both films and the ionization chamber are listed in this table. The percentage differences between EBT film values and Semiflex ionization chamber values are calculated according to formula 5:

**Table 5:** NOD obtained from skin dosimetry in RAO and RPO of parotid cancer radiotherapy for EBT and EBT3 radioChromic films and the percentage dose differences (%). The film positions are based on Figure 3.

**Film Position**	**RAO Field**			**RPO Field**		
	**NOD**		**Diff. (%)**	**NOD**		**Diff. (%)**
	**EBT**	**EBT3**		**EBT**	**EBT3**	
1	0.0352	0.0285	19.04	0.0491	0.0367	25.25
2	0.0390	0.0329	15.64	0.0415	0.0329	20.72
3	0.0434	0.0333	23.27	0.0437	0.0333	23.80
4	0.0440	0.0313	28.86	0.0431	0.0335	22.27
5	0.0433	0.0345	20.32	0.0495	0.0365	26.26

The percentage differences between the values by EBT3 film and Semiflex ionization chamber were calculated from a similar equation to formula 5.

Percent Difference(%)=((EBT dose-ionization chamber dose)/EBT dose)×100)           (5)

### Skin dose measurement in parotid cancer treatment

NOD and the percentage difference between NOD from Gafchromic EBT and EBT3 films for RAO and PRO fields in measurement of skin dose in parotid cancer radiotherapy are listed in Table 5. Dosimetry results from using Gafchromic EBT and EBT3 films to measure skin doses for parotid cancer radiotherapy in RAO and PRO radiation fields and also the percentage difference between the values of dose from these two kinds of films are expressed in Table 6.

## Discussion

In this study EBT and EBT3 films were compared with each other in real clinical conditions for skin dose measurement in parotid cancer radiotherapy. According to the data presented in Table 2, it can be observed that in calibration conditions NOD from EBT is a little higher than EBT3 film. The minimum difference between the two series of values was equal to 2.92% which is related to 0.2 Gy dose and the maximum difference is equal to 35.47% which is related to 0.75 Gy dose. Considering that all irradiation and reading conditions for the two films were equal, it can be concluded that this difference is due to the thickness difference of the active layers in these two films. Total thickness of the two active layers in EBT film is 34 micrometer whereas the thickness of one active layer of EBT3 film is 30 micrometer. Since EBT film has a thicker active layer, its sensitivity is greater and this leads to a higher NOD.

According to reported values of NOD in Solid Water phantom, we can observe from Table 3 that the NOD from EBT film in the stated depths is higher than the results from EBT3 film. The minimum difference between the NOD values of the two films in the surface (depth of 0 millimeter) is 1.89 % and the maximum difference (in the depth of 1 mm) is 12.08 %. The larger amount of NOD from EBT film can be related to the higher thickness of the active layer of EBT film compared to EBT3. According to the data given in Table 4, surface dosimetry results reveal that EBT film shows 12.38% dose higher than EBT3 film which is because of larger thickness of EBT film’s active layer.

**Table 3:** NOD obtained from in-phantom measurements for EBT and EBT3 radioChromic films and the percentage dose differences (%)

**Depth (cm)**	**NOD**		**Diff. (%)**
	**EBT**	**EBT3**	
0	0.0317	0.0311	1.89
0.1	0.0861	0.0757	12.08
1.5	0.1475	0.1328	9.97
2.0	0.1442	0.1339	7.14
4.0	0.1354	0.1236	8.71
10.0	0.1064	0.0985	7.42

**Table 4:** Dose (Gy) obtained from EBT and EBT3 radioChromic films and Semiflex ionization chamber and the percentage dose differences (%)

**Depth** **(cm)**	**Dose** **(EBT)**	**Dose** **(EBT3)**	**Dose** **(Semiflex)**	**Diff. (%)** **EBT-EBT3**	**Diff. (%)** **EBT-Semiflex**	**Diff. (%)** **EBT3-Semiflex**
0	0.3159	0.2768	0.61	12.38	-93.10	-120.38
0.1	0.6856	0.7030	0.80	-2.54	-16.69	-13.80
1.5	1.3909	1.3956	1.46	-0.34	-4.97	-0.05
2.0	1.3460	1.4108	1.45	-4.81	-7.73	-2.78
4.0	1.2298	1.2715	1.33	-3.39	-8.15	-4.60
10.0	0.8873	0.9581	1.00	-7.98	-12.70	-4.37

The minimum and the maximum difference between EBT film and Semiflex chamber is 4.97% at depth of 15 mm and 16.69% at the depth of 1 mm.  Additionally, regarding the EBT3 film and Semiflex ionization chamber, the minimum difference at the depth of 15 mm is 0.05% and the maximum difference at depth of 1 mm is 13.80%. Semiflex ionization chamber shows higher dose values than the Gafchromic EBT and EBT3 films in all cases. The diameter of the sensitive volume of Semiflex ionization chamber is 5.5 mm. In surface dosimetry with an ionization chamber part of the sensitive volume is located outside the phantom. This set up is different than that with radioChromic film dosimetry (with 234 µm sensitive layer for EBT and 280 µm for EBT3). The Semiflex ionization chamber has shown 93.10% and 120.38% higher dose in surface measurements compared to EBT and EBT3 films, respectively. In the other depths, the Semiflex ionization chamber has a relatively good agreement with these two films. The difference between the obtained surface doses is due to different thicknesses and thus this ion chamber is not suitable for measurement of surface dose. According to the International Commission on Radiological Protection (ICRP) and International Commission on Radiation Units (ICRU) recommendations, superficial dose or skin dose must be evaluated in the depth of 70 µm[15].

According to the reported values in Table 5, the EBT film shows higher NOD than the EBT3 film for both RAO and RPO fields. The minimum difference in RAO field measurements is observed in position 2 in the field which is equal to 15.64% and the maximum difference between the NOD values of the two films in this field is equal to 28.86% which is related to position 4 in RAO field. The minimum difference of these values for the RPO field is equal to 20.72% which is related to position 2 and the maximum difference is equal to 26.26% in position 5 of this field. While the irradiation and reading conditions were similar for both films. The EBT film has demonstrated higher NOD values due to its thicker active layer.

Moylan et al[7] investigated the accuracy in dosimetry with Gafchromic EBT and EBT3 films and the dependency on the size of the films, region of interest (ROI), and the position of the films on the scanner bed for 6 MV photon beam and 9 MeV electron beam. In that study no increase in uncertainty in dose was observed when the size of the film was reduced. Both EBT2 and EBT3 films had similar fitting functions according to the above conditions, but the Newton fringes artifact for the EBT3 film was less. It was suggested that the film be scanned on the top of the scanner bed. Another finding of that study was that there is no significant difference in dosimetry accuracy between film sizes of 5×5 mm^2^ to 40 × 40 mm^2^. Based on their results, it was suggested that the films be scanned on top of the scanner’s bed but in this study the films were scanned on the bottom of the scanner’s bed during the calibration and measurement process. Mayers et al[16] have suggested that the central part of scanner be used for scanning the films. Various studies have reported different strategies on the selection of the orientation in the scanning step. This subject would benefit from further investigation but may not warrant a full independent research project without further areas of interest. To illuminate this subject, more evaluation with this regard may be useful.

According to Table 6 it can be observed that the dose value from EBT film is always higher than the EBT3 dose value. In the RAO radiation field, the minimum difference between the two films is 15.99% in position 2 in the field and the maximum difference is equal to 25.49% in position 4 in the field. In the RPO radiation field, the minimum difference between the dose values from these two types of films is equal to 18.97% which is related to position 2 of this field and the maximum difference is 20.55% in position 3 of this field. The dose difference between these two films is related to the difference of the fitting functions to the calibration curve.  Different fitting functions for the calibration curves of the films were used to reduce the fitting errors in the calibrations of the films. For fitting the EBT a power function was used and an exponential function was applied for EBT3. In a research paper by Brown et al[11], they investigated the dose response curve of EBT, EBT2, and EBT3 radioChromic films exposed by mono energy X-rays of a synchrotron. The sensitivity of EBT and EBT2 radioChromic films showed large energy dependencies for energies ranging from 25 keV to 4 MV. The magnitude of the energy dependency was less as dose increased. EBT3 film has shown a weak dependency on energy which makes it a more suitable dosimeter for X-rays in keV energy range. Chełmiński et al[17] investigated the Gafchromic EBT film dependency on energy. It was reported that the relative difference exceeds 20% for doses less than 1 Gy but for doses more than 1 Gy this amount becomes 5%. It is suggested that EBT film be used for dose measurements at more than 1 Gy. Uncertainty of more than 20% for doses less than 1 Gy is in agreement with the results of this study.

**Table 6:** Dose (Gy) obtained from skin dosimetry in RAO and RPO of parotid cancer radiotherapy for EBT and EBT3 radioChromic films and the percentage dose differences (%)

**Film Position**	**RAO Field**			**RPO Field**		
	**Dose (Gy)**		**Diff. (%)**	**Dose (Gy)**		**Diff. (%)**
	**EBT**	**EBT3**		**EBT**	**EBT3**	
1	0.3299	0.2546	22.83	0.4026	0.3254	19.18
2	0.3478	0.2922	15.99	0.3606	0.2922	18.97
3	0.3706	0.2957	20.21	0.3722	0.2957	20.55
4	0.3738	0.2785	25.49	0.3690	0.2974	19.40
5	0.3701	0.3061	17.29	0.4055	0.3236	20.20

In the current study, both types of films were scanned in portrait direction. According to our observations, in portrait direction EBT film shows higher NOD than EBT3 film. The pieces of both films were scanned in transmission mode. After film scanning the red channel data in the RGB images was extracted via software processing. In a report done by Papaconstadopoulos et al[18], in which the film pieces were irradiated in the range of  0-8 Gy and were scanned in two reflective and transmission modes in red and green channels, it was observed that EBT3 film shows higher sensitivity when it is scanned in reflective mode and red channel. This condition was about 150% more than when scanning in transmission mode and red channel. It is suggested that other studies be performed in order to compare the magnitude of sensitivity of EBT and EBT3 films to scanning orientation.

Dosimetry using a radioChromic film is an easy and fast method to determine dose distribution using a film sheet[19]. It is very effective for use in relative surface dosimetry[19]. Evaluation of skin dose during the process of treatment planning and the exact measurement of skin dose are important considerations for investigating the skin side effects following radiotherapy[15]. In this study, film dosimetry in RAO and RPO fields was evaluated separately. On the other hand, the total skin dose from two fields in parotid cancer radiotherapy is of clinical importance. Therefore, it is suggested that the skin dose from addition of doses from both radiation fields (RAO, RPO) for parotid cancer radiotherapy be measured by radioChromic film as a future study in this field.

## Conclusion

In calibration conditions, dose measurement at various depths and skin dose measurement for parotid cancer radiotherapy, the EBT film with higher thickness of active layer, showed higher NOD and dose compared with EBT3 film, which contains a 30 micrometer active layer. According to our investigations, EBT film sensitivity has been higher than that of the EBT3 film. The dose differences in small dose ranges for dose measurement in different depths of Solid Water phantom and the skin dose in parotid cancer radiotherapy by using Gafchromic EBT and EBT3 films are partially due to the differences of fitting functions in the calibration curves. Dosimetry results by Gafchromic EBT and EBT3 films show a large difference for surface measurements compared with Semiflex ionization chamber. These films were previously confirmed for measurement of skin dose[14] and based on the results of this study with a relatively high thickness of ionization chamber, it can be concluded that an ionization chamber is not suitable for superficial dosimetry.
